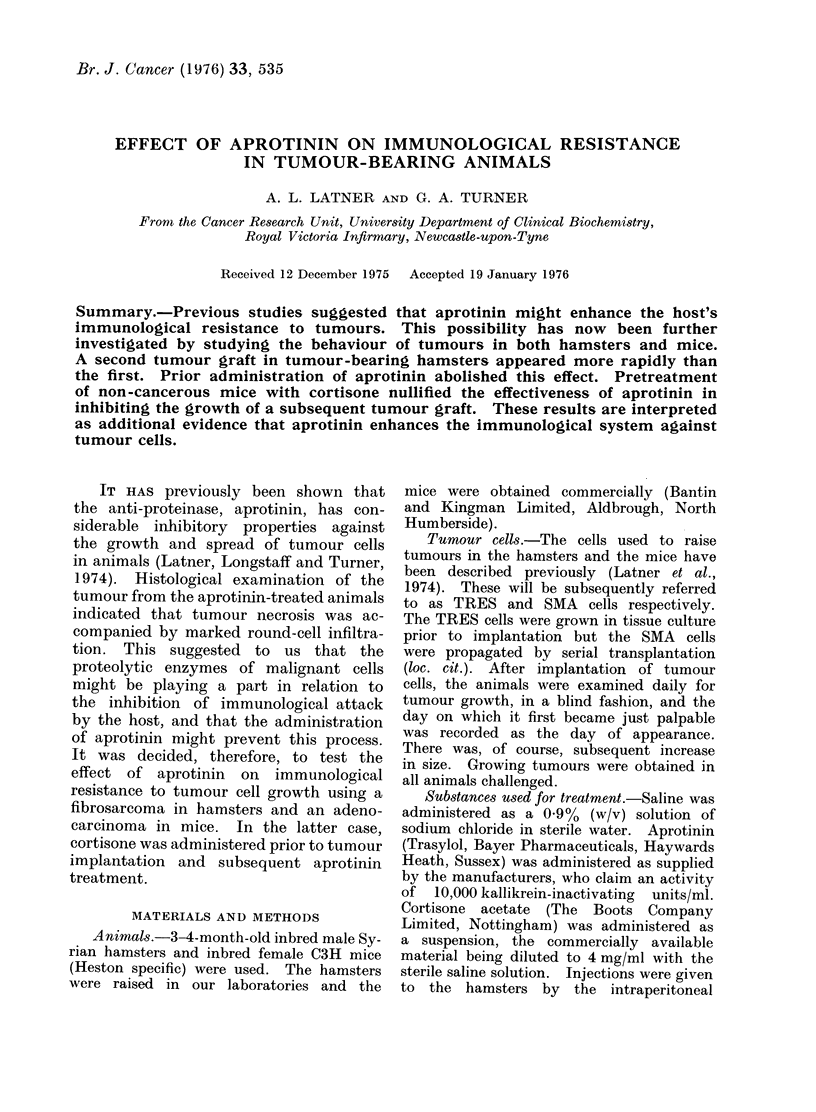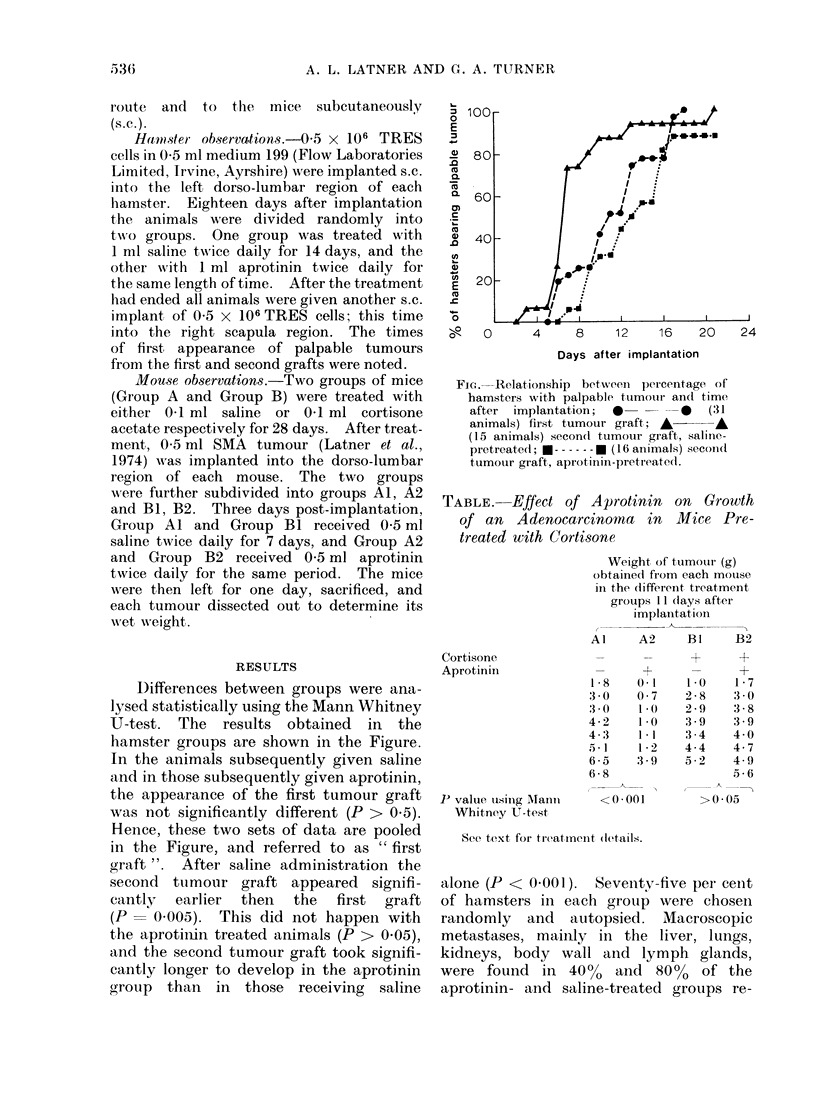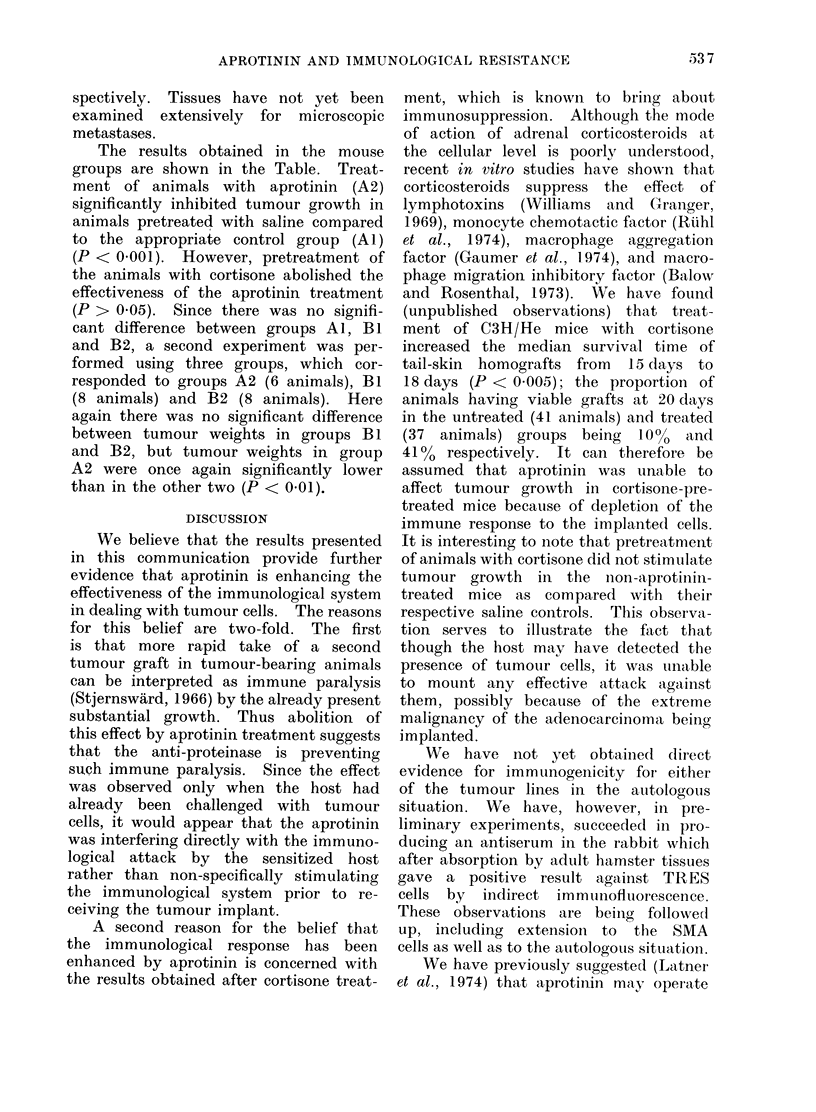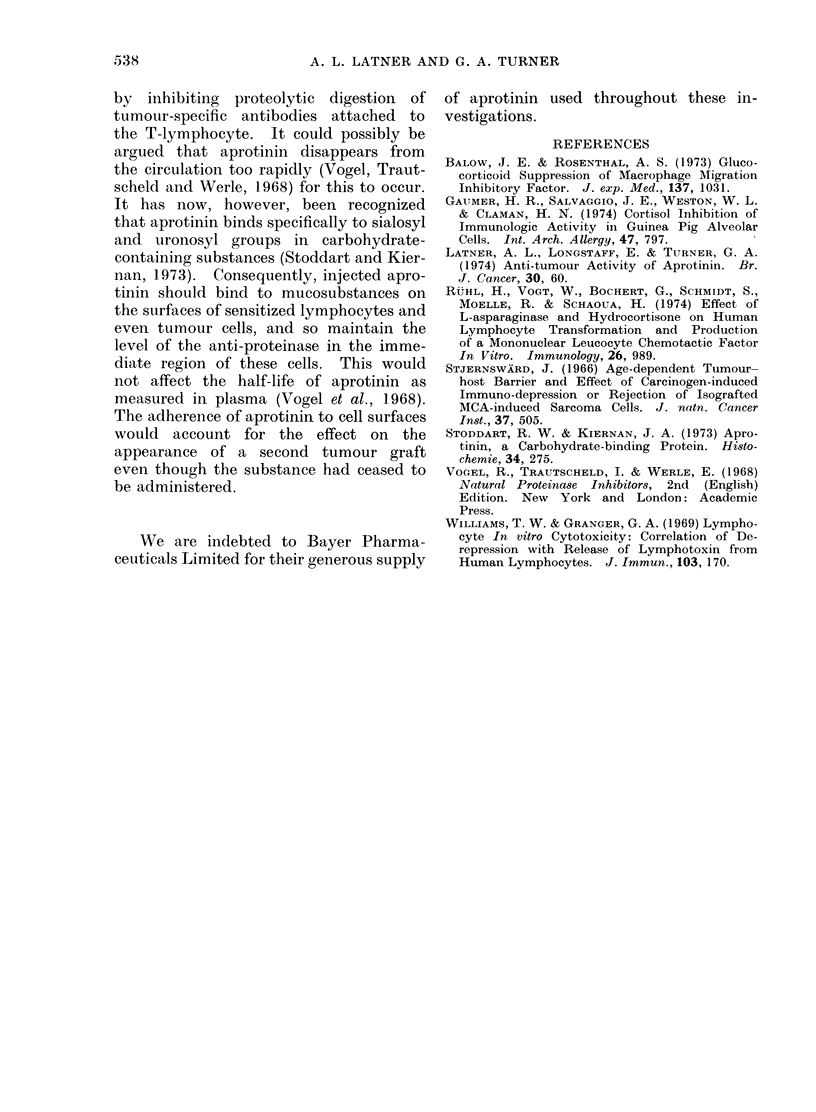# Effect of aprotinin on immunological resistance in tumour-bearing animals.

**DOI:** 10.1038/bjc.1976.84

**Published:** 1976-05

**Authors:** A. L. Latner, G. A. Turner

## Abstract

Previous studies suggested that aprotinin might enhance the host's immunological resistance to tumours. This possibility has now been further investigated by studying the behaviour of tumours in both hamsters and mice. A second tumour graft in tumour-bearing hamsters appeared more rapidly than the first. Prior administration of aprotinin abolished this effect. Pretreatment of non-cancerous mice with cortisone nullified the effectiveness of aprotinin in inhibiting the growth of a subsequent tumour graft. These results are interpreted as additional evidence that aprotinin enhances the immunological system against tumour cells.


*Br. J. Cancer* (1976) **33**, 535

### EFFECT OF APROTININ ON IMMUNOLOGICAL RESISTANCE IN TUMOUR-BEARING ANIMALS

A. L. LATNER AND G. A. TURNER

*From the Cancer Research Unit, University Department of Clinical Biochemistry, Royal Victoria Infirmary, Newcastle-upon-Tyne*

Received 12 December 1975 Accepted 19 January 1976

**Summary.—Previous studies suggested that aprotinin might enhance the host's immunological resistance to tumours. This possibility has now been further investigated by studying the behaviour of tumours in both hamsters and mice. A second tumour graft in tumour-bearing hamsters appeared more rapidly than the first. Prior administration of aprotinin abolished this effect. Pretreatment of non-cancerous mice with cortisone nullified the effectiveness of aprotinin in inhibiting the growth of a subsequent tumour graft. These results are interpreted as additional evidence that aprotinin enhances the immunological system against tumour cells.**

IT HAS previously been shown that the anti-proteinase, aprotinin, has considerable inhibitory properties against the growth and spread of tumour cells in animals (Latner, Longstaff and Turner, 1974). Histological examination of the tumour from the aprotinin-treated animals indicated that tumour necrosis was accompanied by marked round-cell infiltration. This suggested to us that the proteolytic enzymes of malignant cells might be playing a part in relation to the inhibition of immunological attack by the host, and that the administration of aprotinin might prevent this process. It was decided, therefore, to test the effect of aprotinin on immunological resistance to tumour cell growth using a fibrosarcoma in hamsters and an adenocarcinoma in mice. In the latter case, cortisone was administered prior to tumour implantation and subsequent aprotinin treatment.

### MATERIALS AND METHODS

*Animals.*—3–4-month-old inbred male Syrian hamsters and inbred female C3H mice (Heston specific) were used. The hamsters were raised in our laboratories and the mice were obtained commercially (Bantin and Kingman Limited, Aldbrough, North Humberside).

*Tumour cells.*—The cells used to raise tumours in the hamsters and the mice have been described previously (Latner *et al.*, 1974). These will be subsequently referred to as TRES and SMA cells respectively. The TRES cells were grown in tissue culture prior to implantation but the SMA cells were propagated by serial transplantation (*loc. cit.*). After implantation of tumour cells, the animals were examined daily for tumour growth, in a blind fashion, and the day on which it first became just palpable was recorded as the day of appearance. There was, of course, subsequent increase in size. Growing tumours were obtained in all animals challenged.

*Substances used for treatment.*—Saline was administered as a 0·9% (w/v) solution of sodium chloride in sterile water. Aprotinin (Trasylol, Bayer Pharmaceuticals, Haywards Heath, Sussex) was administered as supplied by the manufacturers, who claim an activity of 10,000 kallikrein-inactivating units/ml. Cortisone acetate (The Boots Company Limited, Nottingham) was administered as a suspension, the commercially available material being diluted to 4 mg/ml with the sterile saline solution. Injections were given to the hamsters by the intraperitoneal route and to the mice subcutaneously (s.c.).

*Hamster observations.*—0·5 × $10^6$ TRES cells in 0·5 ml medium 199 (Flow Laboratories Limited, Irvine, Ayrshire) were implanted s.c. into the left dorso-lumbar region of each hamster. Eighteen days after implantation the animals were divided randomly into two groups. One group was treated with 1 ml saline twice daily for 14 days, and the other with 1 ml aprotinin twice daily for the same length of time. After the treatment had ended all animals were given another s.c. implant of 0·5 × $10^6$ TRES cells; this time into the right scapula region. The times of first appearance of palpable tumours from the first and second grafts were noted.

*Mouse observations.*—Two groups of mice (Group A and Group B) were treated with either 0·1 ml saline or 0·1 ml cortisone acetate respectively for 28 days. After treatment, 0·5 ml SMA tumour (Latner *et al.*, 1974) was implanted into the dorso-lumbar region of each mouse. The two groups were further subdivided into groups A1, A2 and B1, B2. Three days post-implantation, Group A1 and Group B1 received 0·5 ml saline twice daily for 7 days, and Group A2 and Group B2 received 0·5 ml aprotinin twice daily for the same period. The mice were then left for one day, sacrificed, and each tumour dissected out to determine its wet weight.

### RESULTS

Differences between groups were analysed statistically using the Mann Whitney U-test. The results obtained in the hamster groups are shown in the Figure. In the animals subsequently given saline and in those subsequently given aprotinin, the appearance of the first tumour graft was not significantly different ($P > 0·5$). Hence, these two sets of data are pooled in the Figure, and referred to as " first graft ". After saline administration the second tumour graft appeared significantly earlier then the first graft ($P = 0·005$). This did not happen with the aprotinin treated animals ($P > 0·05$), and the second tumour graft took significantly longer to develop in the aprotinin group than in those receiving saline alone ($P < 0·001$). Seventy-five per cent of hamsters in each group were chosen randomly and autopsied. Macroscopic metastases, mainly in the liver, lungs, kidneys, body wall and lymph glands, were found in 40% and 80% of the aprotinin- and saline-treated groups re-

FIG.—Relationship between percentage of hamsters with palpable tumour and time after implantation; ●— — —● (31 animals) first tumour graft; ▲———▲ (15 animals) second tumour graft, saline-pretreated; ■- - - - - -■ (16 animals) second tumour graft, aprotinin-pretreated.

TABLE.—*Effect of Aprotinin on Growth of an Adenocarcinoma in Mice Pretreated with Cortisone*

| | Weight of tumour (g) obtained from each mouse in the different treatment groups 11 days after implantation | | | |
|---|---|---|---|---|
| | A1 | A2 | B1 | B2 |
| Cortisone | − | − | + | + |
| Aprotinin | − | + | − | + |
| | 1·8 | 0·1 | 1·0 | 1·7 |
| | 3·0 | 0·7 | 2·8 | 3·0 |
| | 3·0 | 1·0 | 2·9 | 3·8 |
| | 4·2 | 1·0 | 3·9 | 3·9 |
| | 4·3 | 1·1 | 3·4 | 4·0 |
| | 5·1 | 1·2 | 4·4 | 4·7 |
| | 6·5 | 3·9 | 5·2 | 4·9 |
| | 6·8 | | | 5·6 |
| *P* value using Mann Whitney U-test | <0·001 | | >0·05 | |

See text for treatment details.

spectively. Tissues have not yet been examined extensively for microscopic metastases.

The results obtained in the mouse groups are shown in the Table. Treatment of animals with aprotinin (A2) significantly inhibited tumour growth in animals pretreated with saline compared to the appropriate control group (A1) ($P < 0{\cdot}001$). However, pretreatment of the animals with cortisone abolished the effectiveness of the aprotinin treatment ($P > 0{\cdot}05$). Since there was no significant difference between groups A1, B1 and B2, a second experiment was performed using three groups, which corresponded to groups A2 (6 animals), B1 (8 animals) and B2 (8 animals). Here again there was no significant difference between tumour weights in groups B1 and B2, but tumour weights in group A2 were once again significantly lower than in the other two ($P < 0{\cdot}01$).

### DISCUSSION

We believe that the results presented in this communication provide further evidence that aprotinin is enhancing the effectiveness of the immunological system in dealing with tumour cells. The reasons for this belief are two-fold. The first is that more rapid take of a second tumour graft in tumour-bearing animals can be interpreted as immune paralysis (Stjernswärd, 1966) by the already present substantial growth. Thus abolition of this effect by aprotinin treatment suggests that the anti-proteinase is preventing such immune paralysis. Since the effect was observed only when the host had already been challenged with tumour cells, it would appear that the aprotinin was interfering directly with the immunological attack by the sensitized host rather than non-specifically stimulating the immunological system prior to receiving the tumour implant.

A second reason for the belief that the immunological response has been enhanced by aprotinin is concerned with the results obtained after cortisone treatment, which is known to bring about immunosuppression. Although the mode of action of adrenal corticosteroids at the cellular level is poorly understood, recent *in vitro* studies have shown that corticosteroids suppress the effect of lymphotoxins (Williams and Granger, 1969), monocyte chemotactic factor (Rühl *et al.*, 1974), macrophage aggregation factor (Gaumer *et al.*, 1974), and macrophage migration inhibitory factor (Balow and Rosenthal, 1973). We have found (unpublished observations) that treatment of C3H/He mice with cortisone increased the median survival time of tail-skin homografts from 15 days to 18 days ($P < 0{\cdot}005$); the proportion of animals having viable grafts at 20 days in the untreated (41 animals) and treated (37 animals) groups being 10% and 41% respectively. It can therefore be assumed that aprotinin was unable to affect tumour growth in cortisone-pretreated mice because of depletion of the immune response to the implanted cells. It is interesting to note that pretreatment of animals with cortisone did not stimulate tumour growth in the non-aprotinin-treated mice as compared with their respective saline controls. This observation serves to illustrate the fact that though the host may have detected the presence of tumour cells, it was unable to mount any effective attack against them, possibly because of the extreme malignancy of the adenocarcinoma being implanted.

We have not yet obtained direct evidence for immunogenicity for either of the tumour lines in the autologous situation. We have, however, in preliminary experiments, succeeded in producing an antiserum in the rabbit which after absorption by adult hamster tissues gave a positive result against TRES cells by indirect immunofluorescence. These observations are being followed up, including extension to the SMA cells as well as to the autologous situation.

We have previously suggested (Latner *et al.*, 1974) that aprotinin may operate by inhibiting proteolytic digestion of tumour-specific antibodies attached to the T-lymphocyte. It could possibly be argued that aprotinin disappears from the circulation too rapidly (Vogel, Trautscheld and Werle, 1968) for this to occur. It has now, however, been recognized that aprotinin binds specifically to sialosyl and uronosyl groups in carbohydrate-containing substances (Stoddart and Kiernan, 1973). Consequently, injected aprotinin should bind to mucosubstances on the surfaces of sensitized lymphocytes and even tumour cells, and so maintain the level of the anti-proteinase in the immediate region of these cells. This would not affect the half-life of aprotinin as measured in plasma (Vogel *et al.*, 1968). The adherence of aprotinin to cell surfaces would account for the effect on the appearance of a second tumour graft even though the substance had ceased to be administered.

We are indebted to Bayer Pharmaceuticals Limited for their generous supply of aprotinin used throughout these investigations.

### REFERENCES

Balow, J. E. & Rosenthal, A. S. (1973) Glucocorticoid Suppression of Macrophage Migration Inhibitory Factor. *J. exp. Med.*, **137**, 1031.

Gaumer, H. R., Salvaggio, J. E., Weston, W. L. & Claman, H. N. (1974) Cortisol Inhibition of Immunologic Activity in Guinea Pig Alveolar Cells. *Int. Arch. Allergy*, **47**, 797.

Latner, A. L., Longstaff, E. & Turner, G. A. (1974) Anti-tumour Activity of Aprotinin. *Br. J. Cancer*, **30**, 60.

Rühl, H., Vogt, W., Bochert, G., Schmidt, S., Moelle, R. & Schaoua, H. (1974) Effect of L-asparaginase and Hydrocortisone on Human Lymphocyte Transformation and Production of a Mononuclear Leucocyte Chemotactic Factor *In Vitro*. *Immunology*, **26**, 989.

Stjernswärd, J. (1966) Age-dependent Tumour–host Barrier and Effect of Carcinogen-induced Immuno-depression or Rejection of Isografted MCA-induced Sarcoma Cells. *J. natn. Cancer Inst.*, **37**, 505.

Stoddart, R. W. & Kiernan, J. A. (1973) Aprotinin, a Carbohydrate-binding Protein. *Histochemie*, **34**, 275.

Vogel, R., Trautscheld, I. & Werle, E. (1968) *Natural Proteinase Inhibitors*, 2nd (English) Edition. New York and London: Academic Press.

Williams, T. W. & Granger, G. A. (1969) Lymphocyte *In vitro* Cytotoxicity: Correlation of Derepression with Release of Lymphotoxin from Human Lymphocytes. *J. Immun.*, **103**, 170.